# The Relationship between COPD and Cardiovascular Disease

**Published:** 2017

**Authors:** Jennifer Quint

**Affiliations:** National Heart and Lung Institute, Imperial College London

People with chronic obstructive pulmonary disease (COPD) have a higher incidence of and mortality from cardiovascular disease, and reducing cardiovascular mortality is an important target area for reducing overall mortality in people with COPD. Huge strides have been made in recent decades in reducing mortality after myocardial infarction (MI) in the general population, however, these improvements may not have been mirrored in all patient groups, such as those with COPD.

Recently, much attention has been paid to investigating how COPD patients present with acute MI and how their management and outcomes compare to people without COPD. The evidence seems to point to an increased risk of death after MI in people with COPD, however it is unclear if this difference is due to COPD itself or if it is due to potentially modifiable factors, such as less aggressive treatment after MI. In a recent review we studied the evidence for differences between COPD and non-COPD patients after MI in terms of presentation, recognition of MI, in-hospital care, secondary prevention and outcomes both in-hospital and in the longer term.

People with COPD have both a higher risk of MI and poorer long term outcomes following MI. Previous studies have also shown that COPD patients are less likely to receive beta-blockers on discharge after an MI and are less likely to receive PCI after a STEMI. Findings for differences in in-hospital mortality have been mixed, with some studies finding higher mortality for COPD patients and some studies finding no difference. The heterogeneity in findings may be due to differences in treatment practices. The extent to which differences in treatment can explain differences in mortality at the population level, the “mortality gap”, is unclear.

Delayed diagnosis, timing and use of reperfusion of a STEMI, use of angiography after a non-STEMI, and use of secondary prevention medicines are all potential explanations for the mortality gap after MI in people with COPD. In a recent study, we have found that differences in potentially modifiable in-hospital processes may explain some of the mortality gap between COPD and non-COPD patients after an MI. Clinicians need to be aware that it may be easier to miss MIs in people with COPD and may need to be aware of more unusual presentations of MI in people with COPD. In addition, our results suggest that COPD patients may benefit from more aggressive treatment after an MI.

Troponin is presently used as a prognostic indicator in the context of predicting COPD morbidity and mortality. Acute exacerbations of COPD (AECOPD) are associated with a rise in troponin. Patients with higher troponin have longer hospital stays and worse survival outcome at 1-year. Cardiac Troponin T ≥ 0.04 during AECOPD hospital admission conferred approximately a twice as likely risk of mortality at 1-year than those with non-detectable Cardiac Troponin T. Cardiac Troponin T appears a better prognostic indicator for long-term outcome amongst COPD patients following an MI compared to Troponin I. Clinicians should not be reassured by relative lower troponin levels in COPD patients at the time of an MI.

Despite being at higher risk of death following admission for acute coronary syndromes, people with COPD are less likely to receive investigation and treatment than people without COPD and this difference may explain some of the difference in mortality. It is recommended that those at moderate (3–6%) or high (>6%) Global Registry of Acute Coronary Events (GRACE) score predicted risk of death at 6 months after admission to hospital for non-ST-segment elevation myocardial infarction or unstable angina receive earlier aggressive investigation and treatment. We undertook a nationwide multicentre study involving 481,849 hospital admissions and demonstrated that GRACE scores underestimate risk of death after acute coronary syndromes for those with COPD. This study also found that multiplying the predicted risk of death for those with COPD by 1.3 provides a better approximation for their risk of death. Using a more accurate estimate of risk of death for those with COPD after admission for acute coronary syndromes one third of COPD patients previously categorised as low risk would be reclassified as moderate risk, and therefore would be eligible for earlier, more aggressive investigation and treatment.

